# A meta-analysis of the effects of dual-task training on cognitive function in stroke patients

**DOI:** 10.3389/fneur.2025.1417364

**Published:** 2025-12-22

**Authors:** Ruolin Shi, Weibo Li, Xiaolu Liu, Zengxin Sun, Xinjing Ge, Peiyuan Lv, Yu Yin

**Affiliations:** 1 College of Nursing and Rehabilitation, North China University of Science and Technology, Tangshan, China; 2 Department of Gastrointestinal Surgery, The Second Hospital of Hebei Medical University, Shijiazhuang, China; 3 Department of Rehabilitation Medicine, Hebei General Hospital, Shijiazhuang, China; 4 Hebei Provincial Key Laboratory of Cerebral Networks and Cognitive Disorders, Shijiazhuang, China

**Keywords:** stroke, cognitive dysfunction, post-stroke cognitive impairment, dual-task training, cognition disorders, meta-analysis, randomized controlled trial

## Abstract

**Background:**

Post-stroke cognitive impairment (PSCI) affects ~40% of survivors, hindering recovery. Dual-task training (combining cognitive and motor tasks) may help, but its superiority over single-task training or usual care remains unclear. This study examines whether dual-task training improves cognitive function more than (1) single-task training or (2) usual rehab/control, and whether effects vary by intervention duration.

**Methods:**

Keywords were used to search Chinese and English databases. The search period was up to 15 October 2023. Randomized controlled trial (RCT) studies comparing the effects of dual-task training and single-task training or blank control on improving cognitive impairment in stroke patients were included and the quality of the included studies was evaluated using the Cochrane collaboration’s risk assessment tool. The effect indicators were evaluated based on fixed-effects or random-effects models.

**Results:**

A total of 15 RCT studies were included. The results of the studies showed that there was a significant difference in mini-mental state examination scores in the dual-task training group compared with the control group (*p* < 0.0001). At intervention time >6 weeks trail making test-A scores were lower compared with controls (*p* < 0.00001). After intervention time >4 weeks, there was a significant difference in digit span test-backward scores compared with controls (*p* = 0.0003). There was a significant difference in digit span test-forward scores compared with controls (*p* = 0.0001) after >4 weeks of intervention. There was a significant difference in Montreal Cognitive Assessment scores compared with controls in elderly patients with insignificant cognitive deficits post-stroke (*p* < 0.00001) and patients with significant cognitive impairment following a stroke (*p* < 0.00001).

**Conclusion:**

Dual-task training is more effective than conventional rehabilitation in improving PSCI, but the aspects of improvement may be limited by the duration of the intervention, the number and quality of included studies and the differences in cognitive function, motor tasks and so on.

**Systematic review registration:**

https://www.crd.york.ac.uk/PROSPERO, CRD42023393550.

## Introduction

As one of the common chronic diseases, stroke has an acute onset, progresses rapidly and leaves behind various degrees of functional impairment, bringing a significant burden to the patient’s family and even society (1). Research suggests that around 40% of stroke patients will have cognitive impairment (2). Cognitive dysfunction following a stroke affects progress in rehabilitation of other functions and increases the difficulty of care and rehabilitation at home and in hospitals (3).

Currently, conventional cognitive training and exercise aerobic training are inefficient because they require an exclusive programme tailored to the patient’s own medical characteristics (4). Kumar et al. (5) showed that the transcranial magnetic stimulation technique can help in post-stroke cognitive deficits by modulating the functional areas of the patient’s brain through cortical stimulation, but the therapeutic effect is unstable. Computer-assisted cognitive training and virtual reality technology are not widely used in clinical applications due to their need for a certain amount of capital investment.

Dual-task training is a new training modality applied to stroke patients in recent years, which involves performing one functional training task along with two or more other tasks (6). Studies by Choi et al. (7) and De Luca et al. (8) suggest that a dual task of cognitive–motor training combined with audio synchronization may be more effective in improving attention, cognitive flexibility and executive performance.

Previous systematic reviews have examined dual-task training primarily for its impact on motor outcomes, such as gait parameters (9) or combined motor–cognitive effects (10). These reviews focused on motor function rather than cognitive recovery per se, and none specifically isolated the cognitive benefits of dual-task training versus single-task or usual care in stroke survivors. However, the current study does not analyze single-task training or blank control studies separately to clarify whether the effect of dual-task training is superior to single-task training or to a blank control. The purpose of this study is to investigate whether dual-task training is better than single-task training and blank control in improving cognitive impairment in stroke patients using a meta-analysis. The aim is to provide an evidence-based reference for clinical, dual-task training in the treatment of cognitive dysfunction in stroke patients.

Stroke phase was not an exclusion criterion. Seven of the 15 included trials enrolled participants in the subacute phase (<6 months post-stroke), three recruited chronic patients (>6 months) and five did not specify disease duration. We deliberately retained studies across all phases because (1) the overall literature pool was small and further exclusion would compromise statistical power and generalisability, and (2) our primary objective was to determine whether cognitive–motor dual-task training improves cognitive outcomes relative to usual care or single-task training irrespective of stroke chronicity. Nevertheless, the predominance of participants at a subacute phase should be noted as this window is considered critical for cognitive recovery.

## Methods

### Literature search

The search strategy followed the PRISMA 2020 statement. Computer searches were conducted using the PubMed, Embase, Cochrane Library, Web of Science, China National Knowledge Infrastructure, China Biomedical Literature Database and Wanfang Data databases. The search was available until June 1, 2023, in both Chinese and English. The intervention was dual-task training, the disease type was stroke and the study type was randomized controlled trials (RCTs). The search strategy included both the Chinese and the English languages.

The search terms were as follows: (Strokes OR Cerebrovascular Apoplexy OR Apoplexy, Cerebrovascular OR Cerebrovascular Stroke OR Cerebrovascular Strokes OR Stroke, Cerebrovascular OR Strokes, Cerebrovascular OR Apoplexy OR Cerebral Stroke OR Cerebral Strokes OR Stroke, Cerebral OR Strokes, Cerebral) AND (Cognitions OR Cognitive Function OR Cognitive Functions OR Function, Cognitive OR Functions, Cognitive) AND (dual task OR dual-task OR cognitive task OR cognitive-task OR concurrent task OR cognitive motor OR cognitive-motor OR motor cognitive OR motor-cognitive OR second task OR additional task).

### Inclusion and exclusion criteria

The inclusion criteria were as follows:

1. Participants: Diagnosed with stroke and confirmed by computed tomography or magnetic resonance imaging; age and gender were not limited.
2. Intervention: Dual-task training (including a cognitive task) in the experimental group.
3. Control: Other conventional rehabilitation treatments, also combined with physical physiological factor therapy.
4. Outcome indicators: These included the mini-mental state examination (MMSE) (11), Montreal Cognitive Assessment (MoCA) (11), Trail Making Test-A (TMT-A), Stroop Color and Word Test (SCWT) and Digit Span Test (DST). For details see Table 1 (11–15).
5. Research method: RCT.
6. Language: Chinese and English.

**Table 1:** Outcome indicators: all trials had to report at least one of the following validated cognitive scales.

Scale	Domains assessed	Score range/cut-off	Interpretation	References
Mini-Mental State Examination (MMSE)	Orientation, memory, attention, language, visuospatial	0–30; < 24 indicates cognitive impairment	Global cognitive screening	(11)
Montreal Cognitive Assessment (MoCA)	Executive function, memory, language, visuospatial, attention	0–30; < 26 indicates mild cognitive impairment	More sensitive than MMSE for mild deficits	(11)
Trail Making Test-A (TMT-A)	Processing speed, visual attention	Time to completion (seconds); higher = worse	Measures cognitive flexibility and attention	(12)
Stroop Color–Word Test (word sub-task)	Selective attention, inhibition	Seconds or errors; higher = worse	Assesses executive inhibition	(13)
Digit Span Test-Backward (DST-B)	Working memory	0–14 digits recalled; higher = better	Verbal working memory capacity	(14)
Digit Span Test-Forward (DST-F)	Short-term memory	0–16 digits recalled; higher = better	Verbal short-term memory	(15)

The exclusion criteria included the following:

1. Conference papers.
2. Inability to extract valid ending data from the text.
3. Duplicate literature.
4. Systematic reviews.

Seven records were excluded because their full texts could not be accessed. These comprised conference abstracts with image-only PDFs lacking selectable text, subscription-protected journal articles for which our library has no license, one Wanfang Data record with a URL that returned a persistent 404 error, a *Journal of Physical Therapy Science* paper with an online appendix hosting the required data that was no longer available and two Korean conference papers that were only available in print.

### Literature screening and data extraction

Two investigators independently screened the literature, extracted data and cross-checked against the inclusion and exclusion criteria. If disagreements emerged, they were resolved through discussion or negotiation with a third party. Data extracted in this meta-analysis included title, first author, year of publication, sample size, intervention, duration of intervention and relevant outcome indicators.

### Literature quality evaluation

Two researchers independently assessed the risk of bias of the included studies using the Cochrane-recommended RCT risk of bias assessment tool and cross-checked the results. The evaluation items of the tool include the following seven areas: generation of randomized sequences; allocation concealment; blinding of participants and implementers; blinding of outcome assessments; completeness of outcome data; selective reporting of findings; and other sources of bias (other sources of bias items were excluded from this study).

### Statistical analysis

The data included in the study were quantitatively analyzed using RevMan v5.4 software. Successive results in the same units were analyzed using mean difference (MD); in all other cases, standardised mean difference (SMD) was used. Uncertainties are presented as 95% confidence intervals (95%CI). Heterogeneity was assessed using I^2^; for I^2^ ≤ 50%, *p* ≥ 0.1, heterogeneity was small and a fixed-effects model was used; for I^2^ > 50%, *p* < 0.1, a random-effects model was used; and for I^2^ > 75%, *p* < 0.1, heterogeneity was large and sensitivity or subgroup analysis was used. The Egger and Begg tests were used for publication bias. The level of significance *α* = 0.05. The sample size of this study was less than 10 articles, meaning only subjective publication bias analysis was conducted.

## Results

### Literature screening process and results

**Figure 1:**
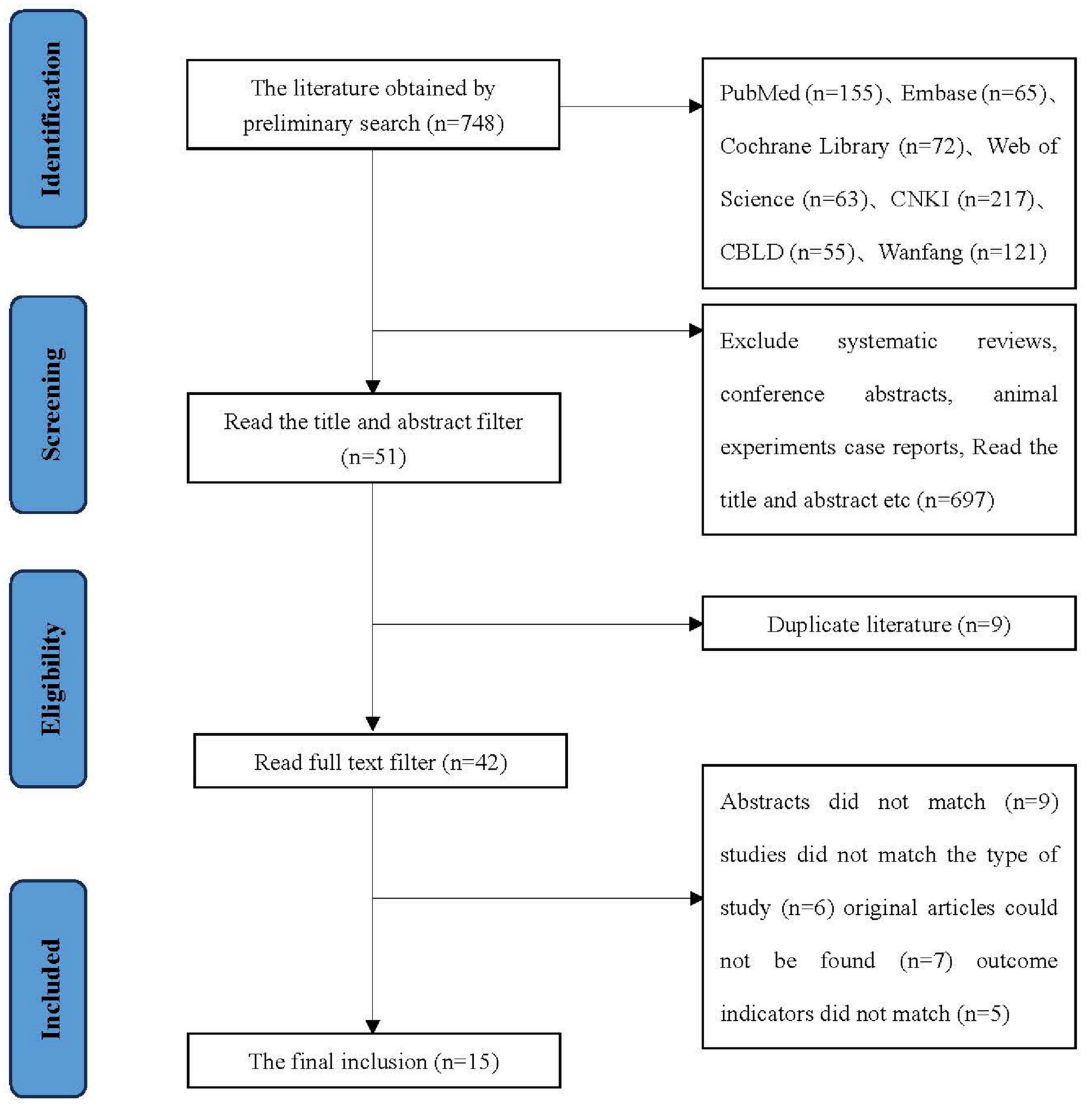
Literature screening process.

A total of 748 pieces of related literature were obtained, and 15 articles were finally included by screening the literature quality, language, type, title, abstract, outcome indicators, duplication or not and access to the original text. The literature screening process and results are shown in Figure 1.

### Basic characteristics of included studies and risk of bias results

**Figure 2:**
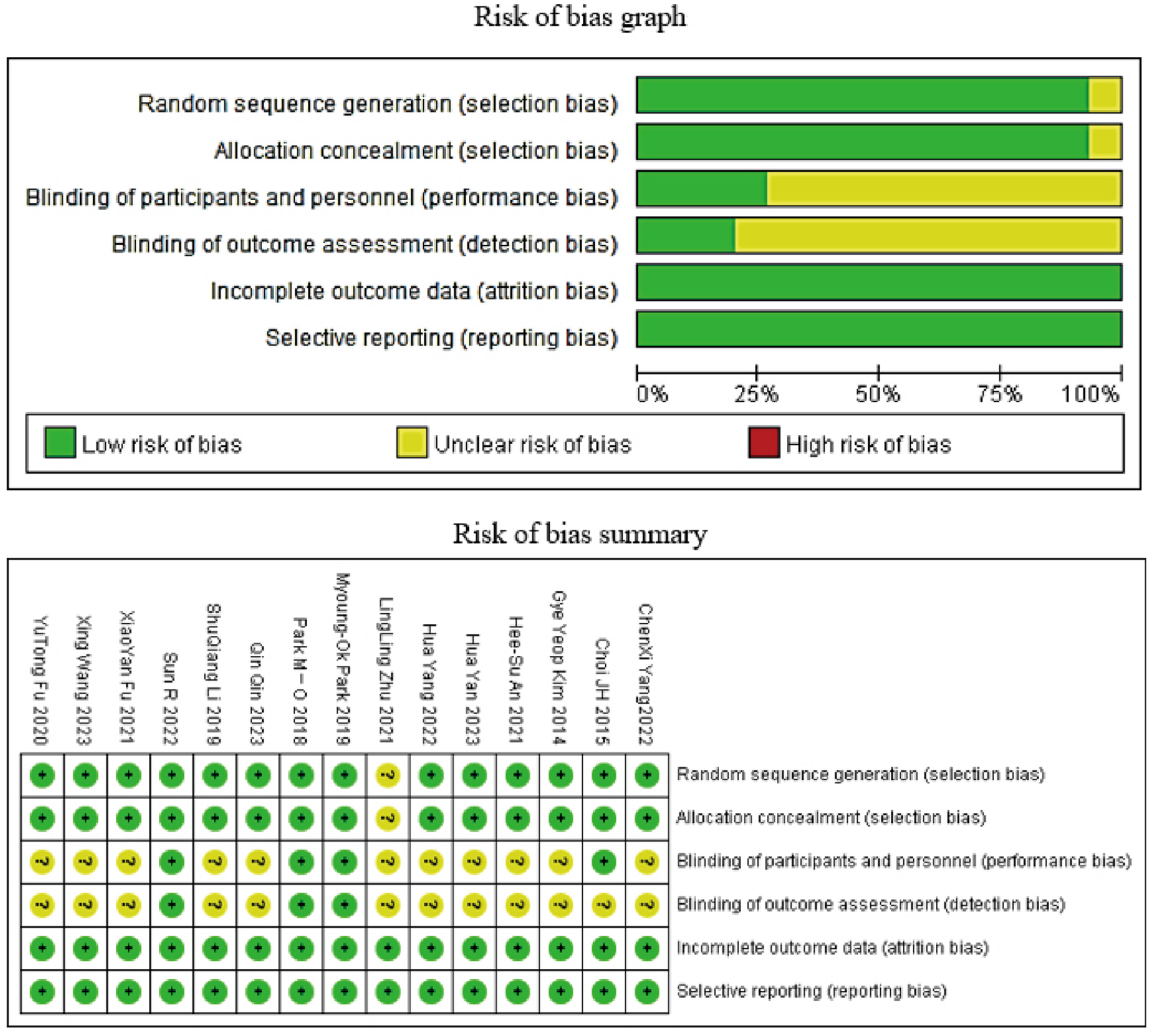
Cochrane risk of bias assessment results map.

The basic characteristics of the included studies are shown in Table 2 (16–30). The Cochrane risk of bias assessment tool was used to evaluate the quality of the literature, with the results shown in Figure 2.

**Table 2:** Table of general characteristics.

Inclusion of studies	Sample size		Intervention		Frequency	Times	Outcome indicator
	EG	CG	CG	EG			
Wang et al. (16)	36	36	Routine rehabilitation and proprioceptive training	Cognitive-motor dual-task training.	40 min/times, 5 times/w	8w	MoCA
Fu et al. (17)	66	65	Repetitive transcranial magnetic stimulation and basic cognitive training	Addition of cognitive-motor control dual-task training to control group treatment	rTMS:15 ~ 20 min, Once a day, 4–5 days a week exercise: Once a day for an hour.	4w	MoCA, Rivermead Behavioural Memory Test (RBMT)
Yan (18)	40	40	Resistance-based rehabilitation	Addition of cognitive-motor control dual-task training to control group treatment	30 min at a time, once a day, 5 times a week	12w	MoCA, Trail Marking Test (TMT)
Yang (19)	99	99	Dual Task Training Therapy	Trained in hyperbaric-assisted dual-tasking	One hour at a time, once a day, 5 times a week	4w	MMSE, MoCA
Yang and Wang (20)	20	20	Routine single-task rehabilitation	Cognitive-motor dual-task training	2 times a day, 5 times a week	2w	MMSE, MoCA, DS, SDMT, TMT-A
Qin et al. (21)	53	53	Routine treatment and routine neurosurgical care	Cognitive-Otago motor control dual task training	90 min a day, once a day, 7 times a week	90d	MoCA
Zhu et al. (22)	38	38	Health education and routine treatment	Perform a simplified version of Otago’s cognitive-motor dual-task training that combines movement and music.	One hour at a time. 2 times per week	90d	MoCA-BJ and Trail Marking Test, (TMT-A)
Fu et al. (23)	15	15	General rehabilitation training such as balance,	Dual-task training	6 times/week, 40–50 min/time.	3w	MMSE
Li et al. (24)	31	31	Exercise Rehabilitation Therapy and Cognitive Rehabilitation Training	Motor rehabilitation therapy and cognitive rehabilitation dual-task training plus AMST training: using an interactive metronome	3 times per week, 30 min/trip.	6w	TMT, Digital Span Test (DST), Stroop test
Kim et al. (25)	10	10	Routine rehabilitation training	Dual-task gait training and cognitive tasks	5 days a week	4 w	Stroop test
Choi et al. (26)	10	10	Balance training with balance boards	Dual Task is simultaneous balance and cognitive training using BioRescue	30 min per day, 5 days per week,	4w	MMSE
Park and Lee (27)	15	15	Only 3 CMDTs per week	Received CMDT + AMST 3 times per week	3 times per week	6w	TMT, DST, Stroop test (ST)
An and Kim (28)	15	15	Perform 20 min of single-task training and receive 10 min of regular occupational therapy	20 min of dual-task training and receive 10 min of regular occupational therapy	30 min each time, 5 times a week	5w	DST-B, DST-F, EFPT-K, K-TMT-e B
Park and Lee (29)	15	15	Traditional occupational therapy	Dual-task training using different cognitive tests	18 interventions of 30 min each, 3 times per week	6w	TMT-A, TMT-B DST-F, DST-B, Stroop test (ST)
Sun et al. (30)	20	20	Individualised multi-disciplinary progressive training programme	Patients in the CMDT group received both cognitive and motor training	Complete 40 min of training per day, 5 days per week	4w	MMSE, MoCA

EG indicates experimental group and CG indicates control group.

### Meta-analyzes results

**Figure 3:**
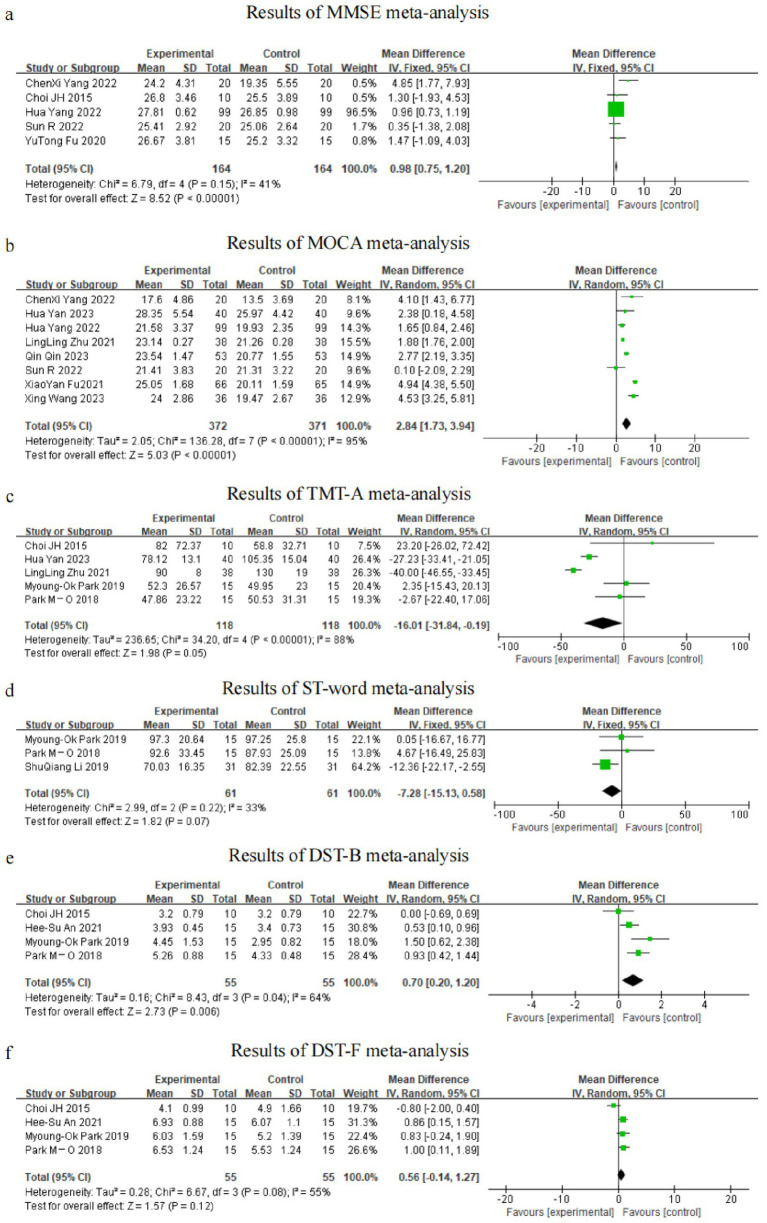
Graph of meta-analysis results **(a)** MMSE, **(b)** MOCA, **(c)** TMT-A, **(d)** ST-word, **(e)** DST-B, **(f)** DST-F.

A total of five RCTs used the MMSE as an outcome indicator, including 328 patients. Since I^2^ = 41%, a fixed-effects model was selected for meta-analysis, which showed better improvement in MMSE scores in the dual-task group relative to the control group (MD = 0.98, 95%CI: 0.75, 1.20, *p* < 0.0001) (see Figure 3a).

A total of eight RCTs used the MoCA as an outcome indicator, including 743 patients. Given that I^2^ = 95%, with large heterogeneity among the results, a random-effects model was used for meta-analysis. The results showed higher scores in the dual-task group compared with the control group (MD = 2.84, 95%CI: 1.73, 3.94, *p* < 0.0001) (see Figure 3b).

A total of five RCTs used the TMT-A trial as an outcome indicator, including 236 patients. Since I^2^ = 88%, with large heterogeneity among the results, a random-effects model was used for meta-analysis. The results showed better improvement in the dual-task group compared with the control group (MD = −16.01, 95%CI: −31.84, −0.19, *p* = 0.05) (see Figure 3c).

A total of three RCTs used the ST-word test as an outcome indicator, including 122 patients. Given that I^2^ = 33%, a fixed-effects model was chosen for meta-analysis, which showed that there was no significant difference in ST-word test scores of the dual-tasking group relative to the control group (MD = −7.28, 95%CI: −15.13, 0.58, *p* = 0.07) (see Figure 3d).

A total of four RCT trials used the DST-backward (DST-B) test as an outcome indicator, including 110 patients. Since I^2^ = 64%, a random-effects model was selected for meta-analysis, which showed that there was a significant difference in DST-B scores of the dual-tasking group relative to the control group (MD = 0.70, 95%CI: 0.20, 1.20, *p* = 0.006) (see Figure 3e).

A total of four RCTs used the DST-forward (DST-F) test as an outcome indicator, including 110 patients. Here, I^2^ = 55%, and a random-effects model was thus used for meta-analysis, which showed no significant difference in DST-F scores of the dual-tasking group relative to the control group (MD = 0.56, 95%CI: −0.14, 1.27, *p* = 0.12) (see Figure 3f).

### Subgroup analysis

**Figure 4:**
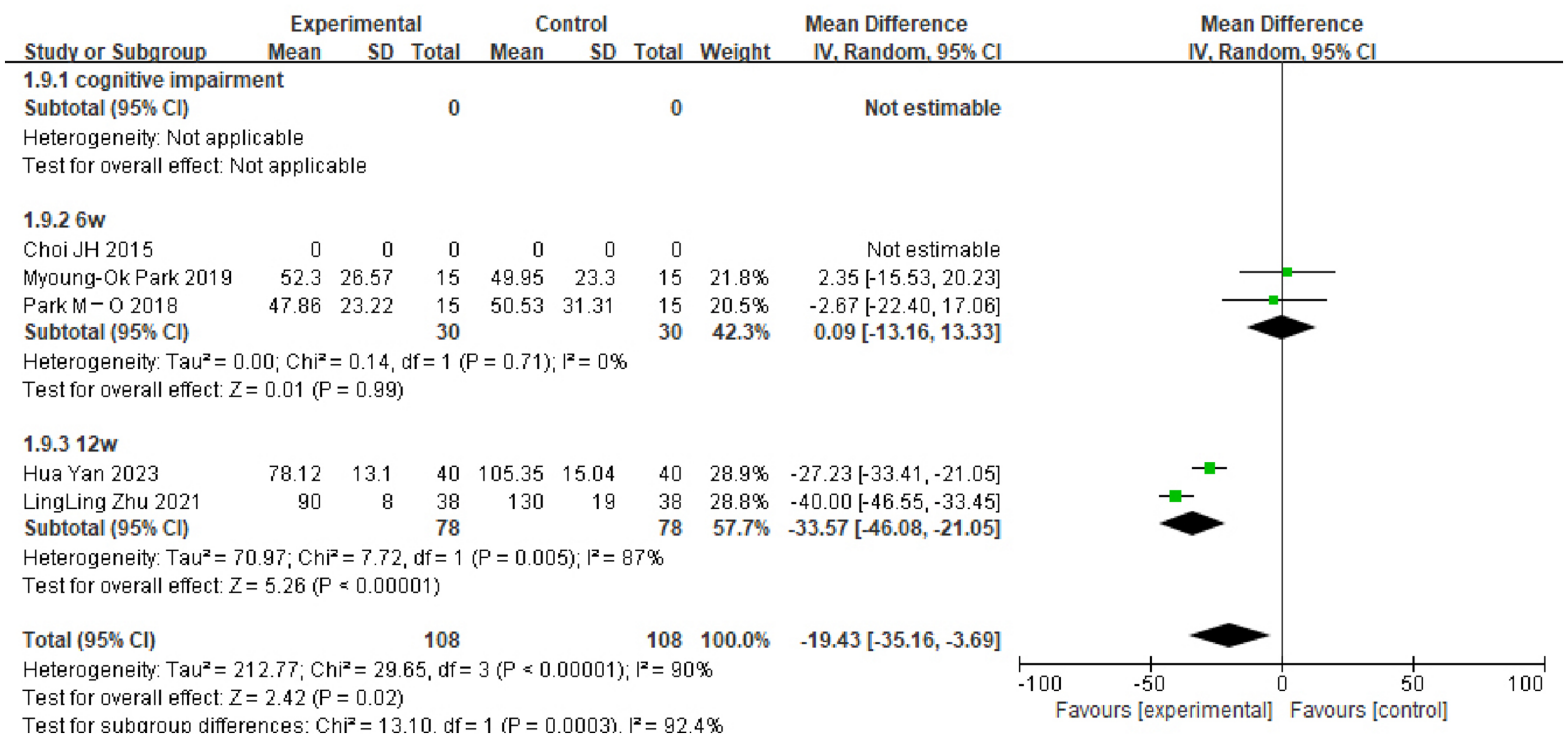
Graph of the results of TMT-A subgroup analyzes.

**Figure 5:**
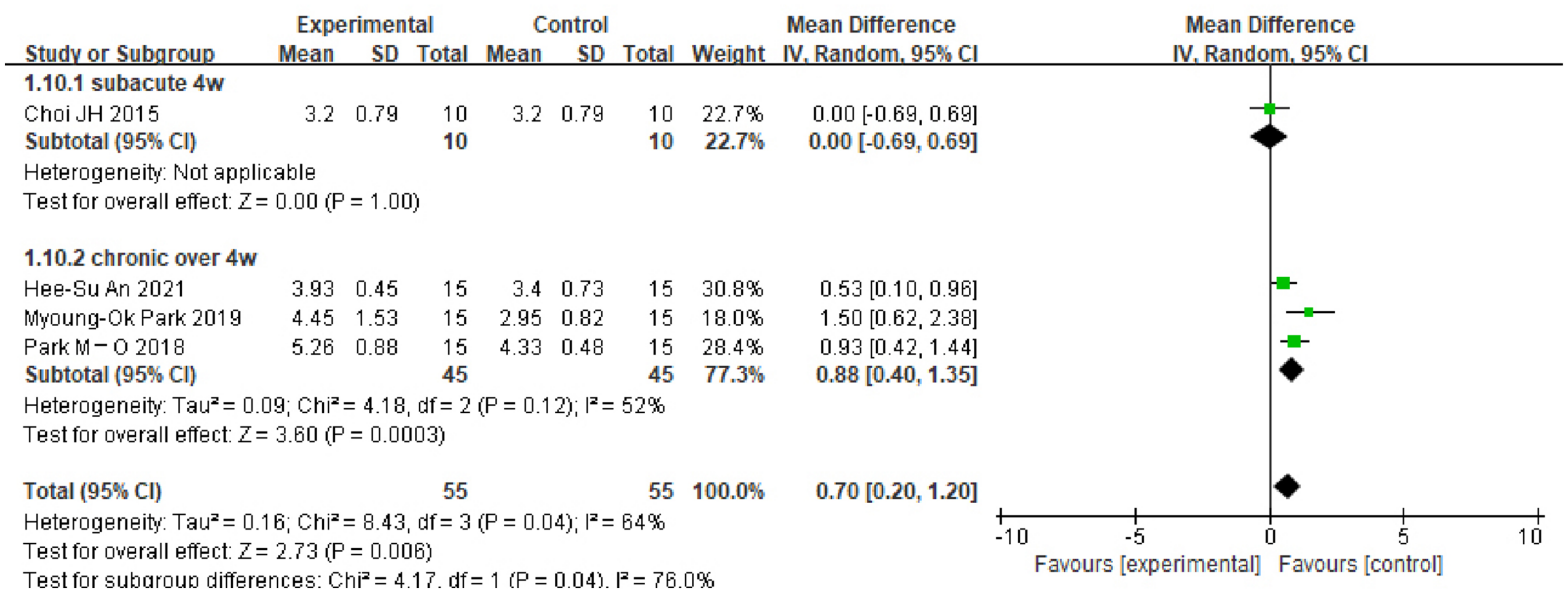
Plot of DST-B test subgroup analysis results.

**Figure 6:**
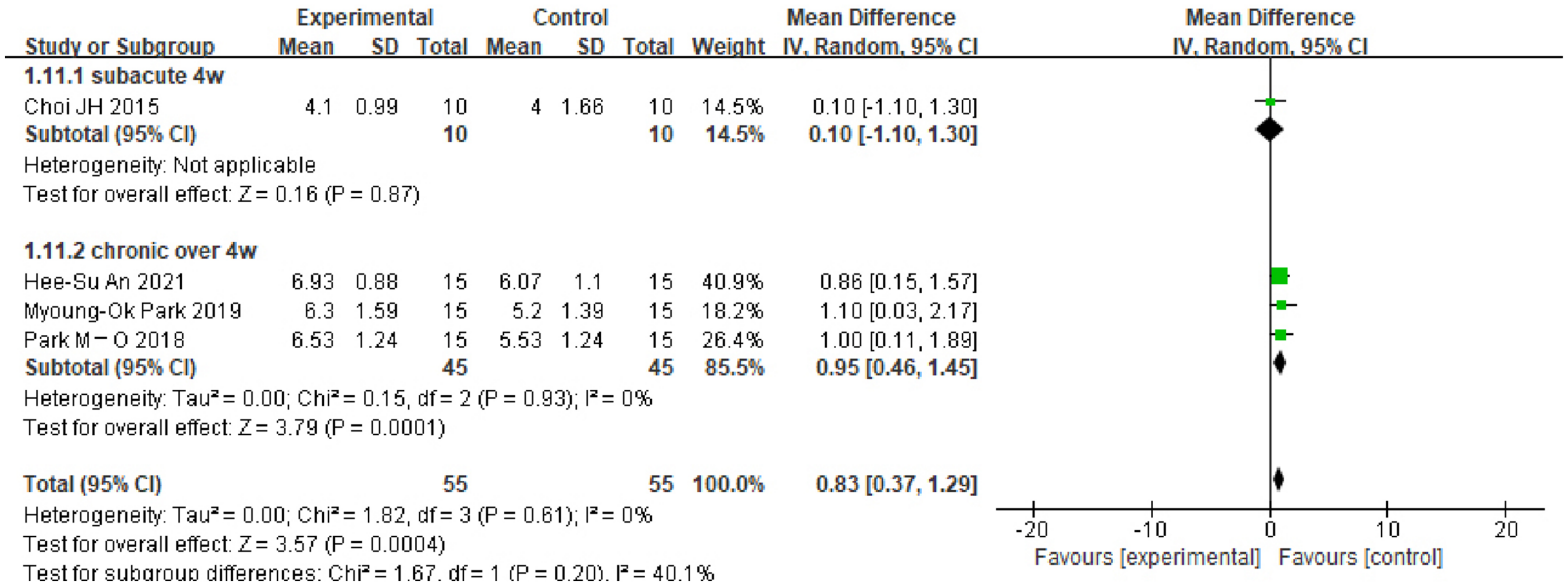
Plot of DST-F test subgroup analysis results.

**Figure 7:**
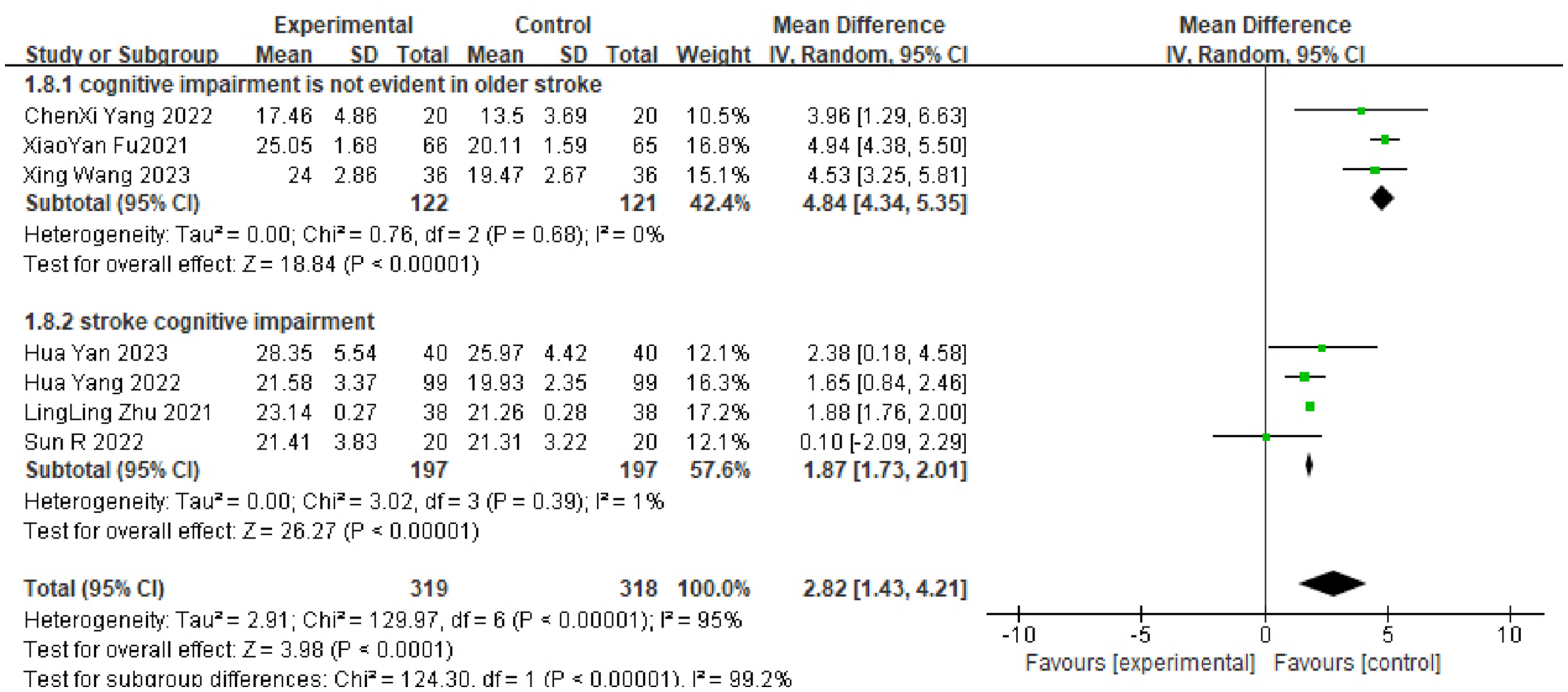
Graph of results of MOCA scale subgroup analyzes.

Due to the high heterogeneity of the results for the MoCA scores (I^2^ = 95%), the TMT-A test (I^2^ = 88%) and the DSTs (I^2^ = 64%, I^2^ = 55%), the sources of heterogeneity were further analyzed to consider possible reasons, such as the type of disease, the degree of cognitive impairment, the duration of the disease and the timing of the intervention. The TMT-A and DSTs were analyzed in subgroups according to the duration of the dual-task intervention, and the MoCA scores were analyzed in subgroups according to the type of disease and the degree of cognitive impairment. The results showed the following. (1) There was no significant difference in TMT-A scores between the dual-task group and the control group at 6 weeks of intervention time (MD = 0.09, 95%CI: −13.16, 13.33, *p* = 0.99), but at >6 weeks of intervention time (MD = −33.57, 95%C1: −46.08, −21.05, *p* < 0.00001), the TMT-A scores were lower compared with the control group (see Figure 4). (2) The dual-task group had a significant difference in DST-B scores compared with the control group after >4 weeks of intervention (MD = 0.88, 95%CI: 0.40, 1.35, *p* = 0.0003) (see Figure 5). (3) The dual-task group had a significant difference in DST-F scores compared with the control group after >4 weeks of intervention (MD = 0.95, 95%CI: 0.46, 1.45, *p* = 0.0001) (see Figure 6). (4) In elderly patients with minor cognitive impairment post-stroke, there was a significant difference in the MoCA score dual-task group compared with the control group (MD = 4.84, 95%CI: 4.34, 5.35, *p* < 0.00001). In patients with significant cognitive impairment post-stroke, the MoCA score was higher in the dual-task group compared with the control group (MD = 1.87, 95%CI: 1.73, 2.01, *p* < 0.00001) (see Figure 7).

### Publication bias

When there are fewer than 10 included studies for meta-analysis of outcome metrics, publication bias analyzes using funnel plots are not recommended, and only subjective publication bias analyzes were, therefore, performed. The small sample size of the included RCTs in this study may have led to a greater risk of publication bias. The inclusion of studies in Chinese and English only, and the exclusion of studies in other languages may also have resulted in some publication bias.

## Discussion

This meta-analysis shows that cognitive–motor dual-task training consistently improves global cognition, executive function and working memory in stroke survivors compared with usual rehabilitation or single-task training, with benefits becoming apparent after 4–6 weeks of intervention.

Post-stroke cognitive impairment (PSCI) is an important cause of long-term disability and reduced quality of life in stroke patients, with approximately half of patients experiencing cognitive impairment in the first year following a stroke (31). Impairments may affect multiple cognitive domains, including information processing, working memory, executive functioning and attention (32, 33). Currently, medication and rehabilitation are the main clinical treatments for cognitive decline associated with PSCI (34). The effects of dual-task training on PSCI were examined. Due to the small sample size and the lack of clarity regarding the results of the intervention, this study aimed to clarify the therapeutic effect of dual-task training on PSCI.

The results of the meta-analyzes showed significant differences in MMSE scores, MoCA scores, TMT-A test scores and DST-B scores in the dual-task group compared with the conventional group. This suggests that cognitive–motor dual-task training is more effective than conventional cognitive training in improving cognitive deficits post-stroke.

The MoCA scale is suitable for screening for mild cognitive impairment post-stroke and has a good ability to detect aspects of executive functioning that are consistent with the cognitive impairment characteristics of PSCI (35). The MMSE is comparable to the MoCA scale for the detection of PSCI, but the MMSE lacks sensitivity for the detection of mild cognitive dysfunction (36) and does not cover a comprehensive enough cognitive domain (37). This study found that both dual-task groups scored better than the control group. It has been shown that the main mechanism of PSCI is caused by stroke leading to lesions such as microhaemorrhage and edema in key areas, such as the hippocampus or cerebral white matter, which causes disruption of neuronal synaptic structure and function in brain regions (38). Park and Lee (27) showed that cognitive–motor dual-task training shortened the reaction time of central nervous system neurons and significantly increased the oxygenation rate of the frontal lobe, thereby improving cognitive performance.

Learning memory impairment is the main symptom of impaired cognitive function post-stroke (39). The DST is commonly used to measure verbal short-term memory and working memory. Studies have applied cognitive–motor dual-task training to the functional training of stroke patients and found that this method not only improved the patients’ walking resistance but also improved executive and memory functions (24, 40). This is in line with the results of the present study, with the DST-F and DST-B scores significantly different in the dual-task group compared with the conventional group after >4 weeks of intervention. The development of cognitive deficits post-stroke is associated with a reduction in the number of synapses and a decrease in the density of connections in hippocampal neurons (41). Dual-task executive function training increases not only hippocampal volume but also cortical area, especially in the prefrontal lobe, through high-intensity training (39).

Executive function is a control mechanism of the brain that includes processes such as planning, initiating, organising, inhibiting, problem solving, self-monitoring and error correction. Approximately 75% of stroke survivors experience executive dysfunction (4). Executive dysfunction reduces the ability to regain independence in activities of daily living. The TMT is a test of executive functioning and attention that focuses on rapid visual search, visuospatial ordering and cognitive orientation transfer. The Stroop Colour and Word Test (SCWT), a widely used measure of executive function, and its operation requires the synergistic action of multiple cognitive functions of the patient, including short-term memory, stereotype switching and attention (42, 43). The effectiveness of conventional cognitive rehabilitation methods in the treatment of patients with executive dysfunction is controversial. Chung et al. (4) found that cognitive rehabilitation intervention was not effective in patients with executive dysfunction. The results of the present study. Showed a difference in TMT-A results compared with the control group at >6 weeks of intervention. Danneels et al. (44) found that dual-task training had an effect on the executive function, memory and visuospatial ability of patients through a dual-task training study combining two forms of postural control with different cognitive tasks, both static and dynamic. This difference may be related to the timing of the intervention, and the specific execution of the cognitive–motor dual-task, including different cognitive activities, with the choice of motor activities also affecting the dual-task training effect due to the different effects of the dual-task interference they create (45, 46). In examining the immediate effects of dual-task obstacle crossing and single-task obstacle crossing training on the functional and cognitive abilities of chronic ambulatory participants with spinal cord injury, Amatachaya et al. (47) found that there was no significant difference in the percentage of errors in SCWT tasks in the dual-task group as compared with the conventional group, which is in line with the results of the present study. Due to the limitations of the number of participants and the duration of the intervention, the results need to be further validated in a large number of clinical trials.

## Conclusion

In summary, cognitive–motor dual-task training is not only a promising adjunct but a clinically superior approach for remediating PSCI when compared with conventional single-task or usual care. The evidence indicates that commencing dual-task programmes within the subacute phase, and continuing them for at least 6 weeks, yields meaningful gains across global cognition, executive function and working memory. These benefits are robust, independent of stroke chronicity and attainable with readily available clinical resources. We therefore recommend that dual-task protocols be routinely integrated into standard stroke rehabilitation pathways and prioritised in future clinical guidelines.

## Data Availability

The original contributions presented in the study are included in the article/supplementary material, further inquiries can be directed to the corresponding author.
